# From energy provision to protein synthesis: Tunnelling nanotubes as mediators of intercellular metabolic cooperation in cancer

**DOI:** 10.1002/2211-5463.70283

**Published:** 2026-06-08

**Authors:** Stanislava Martínková, Jan Trnka

**Affiliations:** ^1^ Laboratory of Metabolism and Bioenergetics, Department of Biochemistry, Cell and Molecular Biology, Third Faculty of Medicine Charles University Prague Czech Republic

**Keywords:** cancer, cytoskeleton, mitochondrial transfer, mRNA transfer, ribosomal transfer, tunnelling nanotubes

## Abstract

Tunnelling nanotubes (TNTs) are thin intercellular membrane structures, which enable direct cytoplasmic communication between distant cells. Since their discovery two decades ago, TNTs have been identified in numerous physiological and pathological contexts. This includes cancer, where they contribute to metabolic cooperation, stress adaptation and treatment resistance. Here we summarise the current understanding of the structural and molecular characteristics of TNTs and their cargoes, including nucleic acids, proteins, organelles, pathogens and drugs. We also discuss the cytoskeletal and motor protein machinery underlying TNT biogenesis and cargo transport. Particular attention is also given to mitochondrial transfer and its role in intercellular metabolic cooperation or parasitism, mRNA transfer and its functional effects in recipient cells, and ribosome transfer which suggests intercellular proteosynthetic cooperation. Overall, while we have learned much about TNTs since their identification a little over 20 years ago, there remain significant questions and discoveries still to be made.

## Graphical abstract

The cytoskeleton‐mediated transport of mitochondria via tunnelling nanotubes restores respiration, increases ATP production, rescues cells from apoptosis, activates the AKT/mTOR signalling pathway, promotes cell migration and invasiveness, contributes to cancer progression and treatment resistance. Recent discoveries demonstrate functional translation of transferred mRNA, phenotypic reprogramming of recipient cells and restoration of protein synthesis driven by intercellular ribosome transfer.

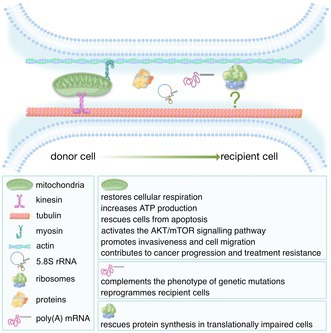

**Abbreviations**
CAD cells: neuronal catecholaminergic cell line derived from Cath.a cells
CAFs: cancer‐associated fibroblasts
ECM: extracellular matrix
EPS8: epidermal growth factor receptor kinase substrate 8
EVs: extracellular vesicles
GFP: green fluorescent protein
GPI: glycosylphosphatidylinositol
IRSp53: insulin receptor tyrosine kinase substrate protein of 53 kDa
iTNTs: individual tunnelling nanotubes
KIF5B: kinesin family member 5B
MHC: major histocompatibility complex
PC12: rat pheochromocytoma cells
TGF‐β: transforming growth factor β
TME: tumour microenvironment
TMs: tumour microtubes
TNFAIP2: tumour necrosis factor‐α‐induced protein 2
TNTs: tunnelling nanotubes

## Introduction: horizontal transfer of cellular material in the tumour microenvironment

Most malignant tumours do not consist solely of malignant cells but are collections of many cellular and noncellular structures, some supporting the malignant growth, others trying to stop it. The tumour microenvironment (TME) is characterised by a complex and heterogeneous ecosystem of tumour, stromal, and immune cells, secreted factors and components of the extracellular matrix (ECM). The ECM is produced largely by cancer‐associated fibroblasts (CAFs), which often arise from resident fibroblasts or stellate cells that become activated into a myofibroblast‐like state. This activation is promoted by factors such as TGF‐β and is associated with extensive ECM deposition, including collagens, proteoglycans and hyaluronan, as well as with the secretion of growth factors and cytokines that support tumour progression [1, 2, 3]. Stromal cells also produce and consume a range of metabolites in metabolic cooperation with cancer cells [4]. The TME can be infiltrated by diverse innate and adaptive immune cells that can exert both tumour‐suppressive and tumour‐promoting functions [5]. Within the tumour microenvironment, cells communicate through soluble molecules, extracellular vesicles, direct cell–cell contacts and long‐range membranous intercellular structures such as tunnelling nanotubes (TNTs) or tumour microtubes (TMs) [6, 7].

The discovery of tunnelling nanotubes two decades ago fundamentally redefined our understanding of direct cell‐to‐cell communication. First described by Rustom and colleagues as thin, membranous, actin‐reinforced tubes forming between rat pheochromocytoma (PC12) cells, human embryonic kidney cells and normal rat kidney cells, TNTs were shown to mediate the intercellular transfer of membrane vesicles and organelles such as endosomes. The presence of the motor protein myosin Va within these structures provided early evidence that TNTs are not passive conduits but dynamic transport systems using cytoskeletal and motor proteins [8].

Since their discovery, extensive research has extended the concept of TNT‐mediated communication to a wide range of physiological and pathological contexts, indicating that TNTs are not restricted to disease but may represent a broader biological mechanism for coordinating cell behaviour during development. In zebrafish gastrulae, TNT‐like connections were shown to transfer cytosolic proteins and early endosomes between cells, whereas in the developing mouse heart, nanotube‐like structures were found to mediate cell–cell contact and the transfer of signalling molecules and proteins; their disruption led to defective myocardial growth, highlighting their importance in cardiac development [9, 10]. Earlier studies had also identified TNT‐like structures in mammalian photoreceptor neurons, primary human proximal tubular epithelial cells and skeletal muscle satellite cells, where they were implicated in the intercellular transfer of cytoplasmic and membrane‐bound material, vesicles and organelles, including mitochondria [11, 12, 13].

In cancer, TNTs have emerged as important mediators of intercellular metabolic cooperation, cellular adaptation and survival under stress conditions. The first evidence for the presence of TNTs in patient‐derived solid tumours was provided by their identification in resected malignant pleural mesothelioma and lung adenocarcinoma samples [14]. TNTs have been shown to facilitate intercellular exchange of cellular RNA, proteins, vesicles and organelles, to support cellular metabolic flexibility and to promote resistance to stress induced by cytotoxic treatments [15, 16, 17, 18, 19, 20, 21, 22, 23, 24, 25, 26, 27, 28]. These findings bring about the notion of TNTs as important regulators of tumour progression and highlight their potential as therapeutic targets to disrupt cellular cooperation within the tumour microenvironment. Unlike other means of intercellular material transfer, such as extracellular vesicles (EVs), where material is released into the extracellular environment and subsequently fuses with recipient cells, TNTs form direct and likely highly regulated connections with specific cells, allowing directional transfer of selected cellular components.

In this review, we summarise the current understanding of structural and molecular characteristics of TNTs, examine the spectrum of molecules and cellular structures exchanged through TNTs in various biological systems including the tumour microenvironment, and focus on mRNA and cellular organelle transfer—primarily mitochondria and ribosomes—as a key determinant of intercellular bioenergetic coupling and protein homeostasis.

## Structural and molecular characteristics of tunnelling nanotubes

Tunnelling nanotubes are thin, actin‐reinforced, open‐ended intercellular membrane tubes that mediate direct cytoplasmic exchange between distant cells [8]. They are typically several hundred nanometres to 100 μm long and form above the cell culture substrate [8, 29]. Their diameter varies depending on the cell type [30]. TNTs can be broadly divided into two categories: thin, short‐lived, actin‐based structures, and thicker, more stable, tubulin‐containing TNTs. Thin TNTs range from 20 to 700 nm in diameter, whereas thick TNTs can exceed 700 nm [8, 31, 32, 33]. Thicker tubulin‐containing TNTs are often induced under stress conditions, such as those present in cancer, and play an important role in the transfer of organelles, including mitochondria [34]. In published glioma studies, particularly those on glioblastoma, the term tumour microtubes has been used to describe long membrane protrusions with a diameter of approximately 1 μm and lengths exceeding 500 μm, which interconnect tumour cells into multicellular networks [6, 7, 35].

Recent advances in high‐resolution imaging have significantly refined our understanding of TNT ultrastructure. A correlative cryo‐electron microscopy study in the neuronal catecholaminergic cell line derived from Cath.a cells (CAD cells) revealed that what often appears to be a single TNT can in fact be a bundle of individual tunnelling nanotubes (iTNTs), each enclosed by its own plasma membrane. Within these iTNTs, vesicles, lysosomes and mitochondria were observed moving unidirectionally through the lumen, while neighbouring iTNTs were frequently aligned in antiparallel orientations. Moreover, thin linkers between adjacent iTNTs were positively labelled for N‐cadherin, suggesting a stabilising role for adhesion molecules in maintaining TNT bundles [36]. The cytoskeletal scaffolding of TNTs is heterogeneous and highly context‐dependent. Actin filaments are considered the canonical structural component of TNTs, but in some cell types, TNTs also contain tubulin. TNTs in PC12 cells were originally described as containing only F‐actin [8], whereas under different culture conditions all TNTs in PC12 cells were later reported to contain both F‐actin and microtubules [37].

Intermediate filament proteins such as cytokeratins have also been shown to contribute to TNT architecture. Longer and more stable nanotubes in the urothelial carcinoma cell line T24 were described as containing both actin and cytokeratin filaments at their bases, but as they elongated, actin filaments were lost, leaving cytokeratin as the defining structural component; in that study, these structures were termed type II TNTs and were distinguished from the shorter and more dynamic actin‐based type I TNTs [38]. Similarly, cytokeratin‐19 has been detected within TNTs in a pancreatic cancer cell line [39] and, more recently, in patient‐derived pancreatic ductal adenocarcinoma biopsy samples expanded in cell culture [28].

Consistent with the distinct structural roles of actin filaments and microtubules in TNT biology, pharmacological studies show that disruption of these cytoskeletal systems produces different functional outcomes. Published studies consistently report that treatment with nocodazole, a microtubule‐depolymerising agent, does not abolish TNT formation, supporting the existence of actin‐based TNTs that can form independently of microtubules [37, 40, 41, 42]. Nevertheless, microtubule depolymerisation induced by agents such as nocodazole or vincristine can impair TNT‐mediated mitochondrial transfer by disrupting microtubule‐dependent transport [41, 43, 44]. In contrast, actin‐targeting agents, including cytochalasin B, cytochalasin D, latrunculin A and latrunculin B, consistently inhibit TNT formation by disrupting actin polymerisation, thereby reducing the intercellular transfer of cytoplasmic cargoes and organelles, including mitochondria, limiting metabolic support and increasing sensitivity of cancer cells to therapy under stress conditions [12, 14, 43, 45, 46, 47]. The disruption of actin polymerisation by latrunculin B or cytochalasin D led to the loss of TNTs and abolished organelle transfer [48]. Similarly, cytochalasin D reduced TNT formation and impaired mitochondrial transfer between primary myeloma cells and bone marrow mesenchymal stromal cells, thereby reducing stromal cell‐mediated drug resistance in multiple myeloma cells [45]. Furthermore, a synthetic migrastatin analogue previously shown to inhibit tumour cell migration, invasion and metastasis by targeting fascin, an actin‐bundling protein involved in the formation of filopodia and other protrusive structures, was later found to suppress TNT formation in malignant pleural mesothelioma cells, together with cancer cell migration [49, 50].

Based on the available literature and our own personal experience, we need to point out that experiments using chemicals directly disrupting the actin cytoskeletal network generally do not allow a direct assessment of the specific role of actin in the transport itself as they often lead to the complete inhibition of TNT formation accompanied by dramatic morphological and functional changes of the cells used in these experiments.

## Formation of TNTs

TNT formation is tightly linked to cell motility and cytoskeletal remodelling, and two mechanistic models have so far been proposed. The first, an actin‐driven model, involves the active extension of a filopodium‐like protrusion from one cell that subsequently fuses with the plasma membrane of another cell, thus establishing a TNT. In the second model, two adjacent cells with intercellular connections move apart and stretch their plasma membranes to generate tethers that subsequently develop into TNTs [34, 38].

Of utmost interest, especially as potential therapeutic targets, are specific molecules allowing the attachment of a growing TNT to the target cells and the fusion of the two cell membranes. Connexin 43 has been implicated in the regulation of TNT formation in breast cancer cells; however, its precise role in TNT formation remains unclear [51]. Available evidence suggests that tetraspanins CD9 and CD81 regulate TNT formation, with CD9 appearing to promote TNT stabilisation, whereas CD81 appears to be required for efficient vesicle transfer through newly formed TNTs, potentially by facilitating interaction with or fusion to the opposing cell in osteosarcoma and neuroblastoma cells [52]. The same study identified in the TNT proteome a number of cell adhesion molecules, which could be candidates for TNT attachment/fusion factors, but the authors note that ‘the TNTome had only a partial overlap with integrin adhesion complexes and with the consensus adhesome’; thus, the function of these proteins in TNTs is unclear. Because the TNTome samples were prepared by mechanical dissociation, it is possible that some components of adhesion complexes remained associated with the cells and were therefore underrepresented in the isolated TNT fraction.

Among the important proteins contributing to TNT biogenesis are the small GTPases RalA, Rac1, and Cdc42, which coordinate M‐Sec/exocyst‐dependent membrane remodelling and actin‐driven protrusion dynamics implicated in TNT formation [42, 53, 54]. M‐Sec (TNFAIP2), a mammalian protein localised to TNT‐like structures, plays a key role in the *de novo* formation of numerous membrane protrusions, and M‐Sec‐induced TNTs were shown to contain actin filaments but not microtubules [53]. Moreover, M‐Sec promoted membrane nanotube formation through interaction with the small GTPase RalA and the exocyst complex [53]. Formation of TNTs also required the activity and differential localisation of small GTPases that are members of the Rho protein family, Cdc42 and Rac1 [16, 42, 55]. Taken together, Ras/Rho GTPase signalling has been shown to be involved in TNT‐mediated intercellular communication, and its inhibition leads to a significant reduction in nanotube formation between immune cells and cancer cells [54], between macrophages [42] and between senescent human primary fibroblasts [56]. Among additional regulators of TNT biogenesis, EPS8 (epidermal growth factor receptor kinase substrate 8) and IRSp53 (insulin receptor tyrosine kinase substrate protein of 53 kDa) have been shown to cooperate in promoting linear actin polymerisation, thereby supporting tunnelling nanotube formation [57]. Upon inhibition of Arp2/3, a complex that initiates the formation of branched actin filaments [58], with CK‐666, the interaction between EPS8 and IRSp53 increased together with TNT formation, supporting a role for EPS8 and IRSp53 in TNT biogenesis through linear actin polymerisation [57].

## Molecular mechanisms of TNT‐mediated transfer

In order to understand how TNT‐mediated intercellular transfer influences cell behaviour, it is essential to uncover the molecular mechanisms of cargo transport through tunnelling nanotubes.

With respect to organelle movement, research has mostly focused on motor proteins and their adaptors. For actin‐based movement the candidate motor proteins are primarily myosins. In mammals, nearly 40 distinct myosin genes have been identified, contributing to diverse cellular processes, including organelle transport, mitosis and cell motility [59]. The earliest description of TNTs identified myosin Va within these structures, pointing to an active, motor‐dependent mechanism of organelle transport rather than passive diffusion [8]. Subsequent studies revealed that the cytoskeletal composition of TNTs determines the directionality and dynamics of transport: actin‐containing TNTs appear to support unidirectional, actomyosin‐dependent trafficking of vesicles and organelles, whereas microtubule‐containing TNTs enable more complex, often bidirectional, cargo movement [32, 60]. Myosin X has been implicated in TNT formation and protrusive behaviour, whereas myosins Va, VI and VIIa have been detected in F‐actin‐containing TNTs, supporting a role for myosin‐based transport of vesicular cargo [61, 62]. Further mechanistic insight came from the identification of myosin XIX (Myo19) as a motor protein that interacts directly with adaptor proteins Miro1/RHOT1 and Miro2/RHOT2 [63].

In addition to actin‐based motors, microtubule‐dependent transport is likely to contribute to cargo movement in thicker TNTs containing tubulin. Here, microtubules may provide tracks for long‐range movement of mitochondria and vesicular cargo, with kinesins generally supporting plus end‐directed transport and cytoplasmic dynein mediating movement towards microtubule minus ends [32, 64]. More recent work in urothelial cells and B‐lymphoma cells further supports this hypothesis, showing that TNTs can contain a composite cytoskeletal architecture comprising actin filaments, intermediate filaments and microtubules, and can associate with motor proteins from the myosin, dynein and kinesin families [65, 66]. Kinesin family member 5B (KIF5B) has been implicated as a microtubule‐based motor for mitochondrial movement through urothelial TNTs [65], and its functional inhibition or knockdown has been shown to reduce mitochondrial transport in cardiomyocyte–myofibroblast and B‐cell lymphoma nanotubes [66, 67].

Molecular motors require adaptor proteins that couple cargo to the cytoskeletal transport machinery. Among the best‐characterised of these are the Miro GTPases of the Rho family, Miro1/RHOT1 and Miro2/RHOT2, which regulate mitochondrial dynamics and link mitochondria to kinesin‐ and dynein‐based transport along microtubules [63, 68, 69, 70]. TRAK adaptor proteins further contribute to this machinery by linking Miro‐associated mitochondria to kinesin‐1 and dynein–dynactin, thereby enabling bidirectional movement along microtubules [71]. Miro1, a mitochondrial Rho GTPase, has been implicated in intercellular mitochondrial trafficking, as its overexpression in mesenchymal stem cells enhanced mitochondrial transfer to epithelial cells and promoted epithelial rescue, whereas its knockdown abolished this protective effect; consistent with this, Miro1 knockdown in carcinoma‐associated mesenchymal stromal cells prevented mitochondrial transfer to ovarian cancer cells and abolished the rescue of proliferation and chemotherapy resistance in mito‐poor tumour cells [72, 73]. Miro1 was further shown to interact directly with a C‐terminal region of the Myo19 tail, thereby supporting mitochondrial transfer in HeLa cells [63]. Collectively, these findings indicate that Miro/RHOT proteins coordinate actin‐ and microtubule‐based mitochondrial trafficking and may also contribute to mitochondrial transfer between cells. In cancer, this concept is further supported by evidence that mitochondrial transfer from cancer cells to fibroblasts through TNTs, both *in vitro* and in xenograft models, depends on Miro2/RHOT2 and promotes the acquisition of a protumorigenic CAF phenotype [74].

## Induction of TNT formation by inflammatory, metabolic and chemotherapeutic stress

The formation of tunnelling nanotubes can be stimulated by a wide range of pathological stressors. It is promoted by pro‐inflammatory conditions, such as lung injury and exposure to Gram‐negative bacterial endotoxins, as reviewed by [75]. A growing body of evidence indicates that viral infection can induce TNT or TNT‐like structure formation in diverse cellular models, suggesting that TNTs may function as a shared stress‐induced mechanism enabling cell‐to‐cell pathogen spread. Studies in macrophages, epithelial cells and placental trophoblasts show that HIV‐1, SARS‐CoV‐2, Zika virus, influenza A and Herpesviruses promote TNT or TNT‐like structure formation, with the common outcome of facilitating intercellular viral dissemination between connected cells [76, 77, 78, 79, 80]. Taken together, TNT‐mediated viral spread may protect viruses from extracellular immune surveillance, neutralising antibodies and antiviral agents, highlighting TNTs as a potentially important therapeutic target and underscoring the need to better understand the mechanisms underlying this mode of intercellular transmission [81].

Metabolic, microenvironmental and treatment‐related stressors are consistently recognised as major drivers of TNT formation across cancer models. In malignant pleural mesothelioma, nutrient deprivation, hyperglycaemia and extracellular acidosis promoted TNT formation together with bidirectional transfer of vesicles, proteins and mitochondria [14], while in prostate cancer both androgen receptor blockade and metabolic stress increased TNT formation in a tumour‐selective manner [82]. Similarly, hypoxia stimulated TNT formation in chemoresistant ovarian cancer cells [83], and oxidative stress increased TNT‐like intercellular connections between astrocytes [84] and, more prominently, in glioblastoma‐associated systems [85]. Taken together, these findings suggest that diverse forms of cellular stress promote a shared response characterised by actin‐dependent membrane remodelling and the increased formation of tunnelling nanotubes.

A similar pattern is observed following exposure to anticancer therapies, supporting the view that TNTs contribute to treatment adaptation. Among cytotoxic agents, doxorubicin induced TNT formation in a dose‐dependent manner in pancreatic cancer cells and facilitated intercellular drug redistribution in both pancreatic and ovarian cancer models [16]. In breast cancer cells, 5‐fluorouracil induced TNT formation and promoted mitochondrial exchange, consistent with a role in survival under treatment stress [86]. In pancreatic ductal adenocarcinoma, even sub‐IC_50_ doses of gemcitabine increased TNT formation [28]. More recently, the effects of chemotherapeutic agents on TNT networks were examined in U87 MG glioblastoma cells; although temozolomide and cytarabine did not significantly alter TNT networks, the study suggested that TNT‐mediated intercellular communication may persist despite treatment with these agents [87]. Together with the studies described above, these observations support the concept that TNTs are stress‐induced conduits that enable cell populations to redistribute drugs and exchange supportive cargo as a rescue mechanism, thereby preserving cell survival. In cancer, this may translate into metabolic plasticity, microenvironmental remodelling and reduced treatment sensitivity, making TNTs a potentially attractive therapeutic target.

## Cargo diversity and selectivity in tunnelling nanotube‐mediated intercellular transfer

Tunnelling nanotubes have been shown to mediate the intercellular exchange of a wide range of cargoes, including nucleic acids, proteins, small molecules, organelles and pathogens. The seminal study by Rustom and co‐authors described the selective transfer of acidic organelles via TNTs in PC12 cells, while small soluble markers such as GFP and the fluorescent chelator calcein were not observed to pass through these structures, indicating a degree of cargo selectivity [8]. Soon after this seminal observation, mitochondrial transfer via TNT‐like connections was observed between endothelial progenitor cells and cardiomyocytes [33], followed by a bidirectional transport of late endosomes and lysosomes in human monocyte‐derived macrophages [32]. Vesicular cargoes have been detected within TNTs in several cellular contexts, including endosomes, endoplasmic reticulum and Golgi components in human monocyte‐derived macrophages, as well as vesicle transfer between stromal cells and chronic myeloid leukaemia cells [62, 76].

Contrary to the original suggestion by Rustom and colleagues that TNTs permit the transfer of larger cellular structures while excluding smaller molecules [8], subsequent studies demonstrated that TNTs can also mediate the intercellular transfer of functionally important membrane proteins, including GPI‐anchored proteins and MHC (major histocompatibility complex) class I molecules in immune cells, as well as MHC class I molecules in HeLa cells [88, 89]. TNTs have been shown to mediate the intercellular transfer of several functionally important proteins, including the multidrug resistance protein P‐glycoprotein in MCF‐7 breast cancer cells and oncogenic KRAS proteins [90, 91, 92]. Accumulating evidence indicates that TNTs are associated with the intercellular presence of ribosome‐related components. Ribosomal proteins were identified in laser‐capture microdissected protrusions from serum‐deprived CAD cells, and more recent studies have reported RNA‐associated ribosomal proteins in TNT‐connected osteosarcoma and pancreatic cancer cells [28, 52, 93]. These studies highlight their potential role in sustaining intercellular translational capacity.

Nucleic acids and pathogenic particles represent another major category of TNT cargoes. MicroRNAs have been shown to transfer between cancer cells [20, 21]. Viral RNA [94, 95], cellular mRNA [23, 25, 28, 96] and ribosomal 5.8S rRNA have also been identified within TNTs [28]. The transfer of mRNA and ribosomes via TNTs [28] and its implications are discussed in detail in section Horizontal transfer of RNA and ribosomes in cancer.

TNT‐mediated pathogen spread has been observed in several contexts, including HIV‐1 spread from infected to uninfected T cells [97]; SARS‐CoV‐2 spread from permissive epithelial cells to nonpermissive neuronal cells [77], and transfer of Zika virus particles together with proteins, mitochondria and RNA to uninfected placental trophoblasts [78]. Similarly, the intercellular spread of prions from bone marrow‐derived dendritic cells to primary neurons via TNTs has also been demonstrated [98]. At the smallest scale, TNTs have also been shown to mediate the intercellular transfer of low‐molecular‐weight cargoes, including ions and chemotherapeutic agents. For example, IP_3_‐evoked Ca^2+^ signals were reported to spread between cultured mammalian cells through TNT connections, and the anthracycline chemotherapeutic doxorubicin was shown to be redistributed via TNTs between connected pancreatic adenocarcinoma cells and between ovarian cancer cells [16, 99].

## Mitochondrial transfer via TNTs modulates energy metabolism in cancer

Mitochondria are evolutionarily distinct, semi‐autonomous organelles with their own genome, essential for aerobic energy metabolism, where oxidative phosphorylation couples electron transfer to ATP synthesis and thereby sustains the cellular ADP/ATP balance [100, 101]. They also contribute to fatty‐acid metabolism, liver ketogenesis, iron–sulphur cluster and haem biosynthesis, redox signalling, calcium homeostasis and apoptosis [102]. Beyond their essential roles in normal and cancer cell metabolism, mitochondria have emerged as transferable organelles of intercellular communication in both healthy tissues and the tumour microenvironment, where their exchange occurs predominantly via tunnelling nanotubes or microtube‐like protrusions and extracellular vesicles, and is generally considered an active, regulated process driven by cytoskeletal remodelling, motor proteins, membrane dynamics and stress‐responsive signalling [103, 104, 105, 106, 107]. A summary of the key studies on intercellular mitochondrial transfer is provided in Table 1.

**Table 1:** Intercellular mitochondrial and mtDNA transfer mediated by tunnelling nanotubes, extracellular vesicles and other routes in cancer and nonmalignant systems. It shows the donor and recipient cells, transferred material, biological system and main functional outcome reported in each study. Overall, the table illustrates that mitochondrial and mtDNA transfer can support metabolic rescue, cellular adaptation, treatment resistance or tissue repair in both cancer and nonmalignant contexts.

Research article	Year	Transport group	Route studied	Donor‐acceptor pair/note	System/field	Donor/recipient cells	Main result
**Cancer**							
(Spees *et al*., 2006) [103]	2006	Not‐defined	Intercellular mitochondrial and mtDNA transfer	Mesenchymal, skin fibroblasts‐to‐human lung adenocarcinoma cells	Lung adenocarcinoma	Human mesenchymal stem cells or human skin fibroblasts‐donor cells; lung adenocarcinoma cells defective or deleted mitochondrial DNA‐ recipient cells	Donor cells transferred mitochondria to respiration‐deficient recipient cells. Transfer restored aerobic respiration and increased ATP levels, establishing mitochondrial transfer as a functional rescue mechanism
(Tan *et al*., 2015) [112]	2015	Horizontal transport	Horizontal acquisition of mtDNA from host cells	Host‐to‐tumour transfer *in vivo*	Melanoma, Breast cancer	Host mouse cells‐donor cells; metastatic murine melanoma and breast carcinoma cells models lacking mitochondrial DNA‐recipient cells	Tumour cells without mtDNA acquired host mtDNA, which restored respiratory function
(Dong *et al*., 2017) [106]	2017	Horizontal transport	Whole‐mitochondria transfer from host	Host‐to‐tumour transfer *in vivo*	Melanoma	Host mouse cells‐donor cells; mtDNA‐deficient mouse melanoma‐recipient cells	*In vivo* rescue of respiration in mtDNA‐deficient tumours, restored tumour‐forming ability
(Pasquier *et al*., 2013) [104]	2013	TNTs	TNT‐mediated mitochondrial and cytoplasmic components transfer	Mesenchymal and endothelial‐to‐ovarian and breast cancer cells	Ovarian tumour, Breast cancer	Bone marrow‐derived mesenchymal cells and endothelial cells‐donor cells; breast and ovarian cancer cell lines‐recipient cells	Transfer of mitochondria occurred preferentially between endothelial cells and cancer cells. Cancer cells acquiring mitochondria displayed chemoresistance
(Wang & Gerdes, 2015) [41]	2015	TNTs	TNT‐mediated mitochondrial transfer	Healthy pheochromocytoma cells‐to‐stressed pheochromocytoma cells	Pheochromocytoma	Untreated pheochromocytoma tumour cells – donor cells; UV‐stressed pheochromocytoma cells‐ recipient cells	Functional mitochondria moved through TNTs into apoptotic/stressed recipient cells. Transfer of functional mitochondria can rescue stressed cells in the early phase of apoptosis
(Lu *et al*., 2017) [46]	2017	TNTs	TNT‐mediated mitochondrial transfer	Highly invasive bladder cancer cells‐to‐less invasive bladder cancer cells	Bladder cancer	Unidirectional transfer of mitochondria from highly malignant urothelial carcinoma cells‐donor cells; less‐invasive bladder cancer cells‐recipient cells; mouse xenograft*/in vivo* invasion models	TNTs promoted unidirectional intercellular mitochondrial transfer from highly invasive urothelial carcinoma cells to less invasive cells derived from a nonmalignant papillary urothelial tumour. The acquired mitochondria fused with the mitochondrial network of recipient cells, increased the invasiveness of the less invasive bladder cancer cells, and activated Akt/mTOR‐related signalling
(Marlein *et al*., 2017) [47]	2017	TNTs	TNT‐mediated mitochondrial transfer	Bone marrow stromal cells‐to‐acute myeloid leukaemia blast	Acute myeloid leukaemia	Bone marrow stromal cells‐donor cells; acute myeloid leukaemia (AML) blast‐ recipient cells; mouse AML models	NOX2‐derived superoxide stimulates mitochondrial transfer from bone marrow stromal cells to AML blasts, thereby conferring a metabolic advantage. Blocking NOX2 reduced mitochondrial transfer, increased AML cell apoptosis, and improved survival in mice
(Marlein *et al*., 2019) [107]	2019	TNTs	TNT‐mediated mitochondrial transfer	Bone marrow stromal cells‐to‐multiple myeloma cells	Multiple myeloma	Bone marrow stromal cells‐donor cells; multiple myeloma cells/malignant plasma cells‐recipient cells/*in vivo* model	Myeloma cells received mitochondria from neighbouring nonmalignant bone marrow stromal cells through tumour‐derived TNTs. This transfer promoted mitochondrial oxidative metabolism and ATP production in recipient myeloma cells, supporting bioenergetic plasticity. CD38 knockdown reduced mitochondrial transfer, decreased mitochondrial respiration, increased apoptosis, slowed disease progression, and improved animal survival
(Pinto *et al*., 2021) [7]	2021	TNTs, TM	TNT‐mediated mitochondrial transfer	Glioblastoma stem‐like cells/ organoid TNT and TM network	Glioblastoma	Patient‐derived glioblastoma stem‐like cells obtained from the external and infiltrative zone of one glioblastoma from one patient; tumour organoids	Glioblastoma stem‐like cells formed functional TNTs and TM in 2D culture and organoids. TNTs enabled mitochondrial transfer and supported tumour network communication
(Valdebenito *et al*., 2021) [85]	2021	TNTs	TNT‐mediated mitochondrial transfer	Glioblastoma cells‐to‐human primary astrocytes	Glioblastoma	Glioblastoma‐donor cells, human primary astrocytes‐recipient cells	Glioblastoma cells transfer mitochondria to surrounding astrocytes via TNTs, reprogramming astrocyte metabolism and increasing their resistance/adaptation to hypoxic tumour conditions
(Matula *et al*., 2021) [45]	2021	TNTs	TNT‐mediated mitochondrial transfer and related direct contacts	Bidirectional mitochondrial transfer between myeloma and bone marrow stromal cells	Multiple myeloma	Bone marrow stromal cells‐donor cells; multiple myeloma cells‐recipient cells	Primary myeloma cells increased mitochondrial acquisition from bone marrow stromal cells in response to chemotherapeutic drugs. Transfer occurred bidirectionally, via TNTs and partial cell fusion, and in myeloma cells it was associated with increased survival and ATP levels together with reduced mitochondrial superoxide levels, supporting drug resistance
(Saha *et al*., 2022) [54]	2022	TNTs	TNT‐mediated mitochondrial transfer	Immune cell‐to‐breast cancer cells	Breast cancer	Immune cells‐donor cells; breast cancer cells‐recipient cells; *in vivo* tumour models	Cancer cells hijacked mitochondria from immune cells through nanotube‐mediated contacts, which was associated with increased basal respiration and spare respiratory capacity in tumour cells grown in coculture. This metabolic advantage in tumour cells was accompanied by depletion of mitochondrial resources in immune cells and a reduction in the immune cell population
(Watson *et al*., 2023) [115]	2023	TM	Contact‐dependent intercellular transfer through GAP43+ membrane connections consistent with TM	Astrocytes‐to‐glioblastoma cells	Glioblastoma	Astrocytes‐donor cells population in the brain microenvironment; glioblastoma cells‐recipient cells; *in vivo*	Acquisition of astrocyte mitochondria increased mitochondrial respiration and ATP production, promoted cell‐cycle progression to G2/M, enhanced self‐renewal, and increased tumour initiation and *in vivo* glioblastoma tumorigenicity
(Goliwas *et al*., 2023) [116]	2023	TNTs	TNT‐mediated mitochondrial transfer	Cancer‐associated fibroblasts‐to‐breast cancer	Breast cancer	Patient‐derived breast cancer‐associated fibroblasts‐donor cells; breast cancer cells‐recipient cells	Transfer of cancer‐associated fibroblasts mitochondria to breast cancer cells increased mitochondrial ATP production with negligible effects on glycolytic ATP production and promoted cancer cell migration/invasion in 3D spheroid models
(Nakhle *et al*., 2023) [118]	2023	TNTs	TNT‐mediated mitochondrial transfer	Mesenchymal stem cells‐to‐glioblastoma stem cells	Glioblastoma	Bone marrow mesenchymal stem cells‐donor cells; glioblastoma stem cells‐recipient cells	Mesenchymal stem cells transferred mitochondria to glioblastoma stem cells through TNTs, leading to metabolic reprogramming and enhanced resistance to temozolomide
(Baldwin *et al*., 2024) [114]	2024	TNTs	TNT‐mediated mitochondrial transfer	Bone marrow stromal cells‐to‐CD8^+^ T cells	Melanoma/leukaemia	Bone marrow stromal cells‐donor cells; CD8+ T cells‐recipient cells; *in vivo* tumour	TNT‐mediated transfer of bone marrow stromal cell‐derived mitochondria to CD8+ T cells enhanced mitochondrial respiration and spare respiratory capacity, thereby improving T‐cell metabolic fitness and promoting superior antitumour responses, which prolonged animal survival
(Cangkrama *et al*., 2025) [74]	2025	TNTs	TNT‐mediated mitochondrial transfer	Cancer cells‐to‐fibroblasts / induction of cancer‐associated fibroblast differentiation	Vulvar cancer, Pancreatic ductal adenocarcinoma, Breast cancer	Vulvar carcinoma cells/Pancreatic ductal adenocarcinoma cells/Breast cancer cells‐donor cells; early passage primary skin fibroblasts‐ recipient cells; *in vitro*, *in vivo*	Functional mitochondria transferred from cancer cells promoted basal respiration and proton leak and increased oxidative phosphorylation and ATP production in recipient fibroblasts. These transferred mitochondria were required to reprogramme fibroblasts into protumorigenic cancer‐associated fibroblasts
(Hoover *et al*., 2025) [119]	2025	TNTs	TNT‐mediated mitochondrial transfer	Neurons‐to‐cancer cells	Breast cancer	Neural stem cell‐derived neuronal cells from the mouse subventricular zone or dorsal root ganglion‐derived neuronal cells‐donor cells; aggressive mouse breast carcinoma cells‐recipient cells; *in vitro*, *in vivo*	Neuron‐to‐cancer mitochondrial transfer upregulated mitochondrial respiration, enhanced spare respiratory capacity, stemness, and resistance to metastatic stressors
(Terasaki *et al*., 2026) [113]	2026	Horizontal transfer/TNTs	Intercellular horizontal/TNT‐mediated mitochondrial transfer	Host immune cells‐to‐cancer cells	Melanoma, Breast cancer, Colon cancer	Host immune cells‐donor cells; melanoma, breast and colon cancer cells‐recipient cells; *in vitro*, *in vivo*	Cancer cells hijacked mitochondria from immune cells, which impaired immune‐cell function. In recipient cancer cells, the transferred mitochondria fused with *in situ* mitochondria in cancer cells, promoted mtDNA leakage into the cytosol, activated cGAS–STING and type I interferon signalling. Mitochondrial transport represents mechanism by which cancer cells evade immunosurveillance and promote colonisation of lymph nodes
(Moschoi *et al*., 2016) [105]	2016	Endocytic	Endocytic mitochondrial transfer	Bone marrow mesenchymal stromal cells‐to‐acute myeloid leukaemia cells	Acute myeloid leukaemia	Bone marrow mesenchymal stromal cells‐donor cells; primary acute myeloid leukaemia blasts/acute myeloid leukaemia cells‐recipient cells; mouse acute myeloid leukaemia models	Acute myeloid leukaemia cells acquired functional mitochondria from bone marrow stromal cells during chemotherapy, leading to increased mitochondrial ATP production. Transfer supported acute myeloid leukaemia better survival and an increased regrowing potential. Mitochondrial transfer was observed *in vitro* and *in vivo* and was reported to occur through endocytic pathways
(Sansone *et al*., 2017) [117]	2017	EVs	EV‐mediated mtDNA transfer via exosomes	Cancer‐associated fibroblasts‐to‐breast cancer cells	Breast cancer	Cancer‐associated fibroblasts‐derived extracellular vesicles carrying the full mitochondrial genome transferred mtDNA‐donor cells; hormonal therapy‐treated or hormonal therapy‐naive breast cancer cells‐recipient cells	Cancer‐associated fibroblasts‐derived EVs carrying the full mitochondrial genome transferred mtDNA to breast cancer cells, thereby restoring oxidative phosphorylation, promoting escape from metabolic quiescence, and contributing to endocrine therapy resistance, particularly in cancer stem‐like cell populations
(Zhou *et al*., 2024) [120]	2024	EVs	EV‐mediated mtDNA transfer	Cancer‐associated fibroblasts‐to‐lung cancer cells	Lung cancer	High‐metastatic lung cancer cell‐derived EVs activate normal fibroblasts into CAFs; tumour‐EV‐activated CAFs then act as mtDNA‐donor cells; lung cancer cells with damaged mitochondria act as‐ recipient cells	Lung cancer cell‐derived EVs activated normal fibroblasts into cancer‐associated fibroblasts, whose conditioned medium then transferred mtDNA to tumour cells with damaged mitochondria. This mtDNA transfer promoted metastasis by restoring mitochondrial function in tumour cells and increasing their resistance to oxidative stress
(Frisbie *et al*., 2024) [73]	2024	Direct contact	Direct mitochondrial transfer	Carcinoma‐associated mesenchymal stem cells‐to‐ovarian cancer cells	Ovarian cancer	Patient‐derived carcinoma‐associated mesenchymal stem/stromal cells‐donor cells; ovarian cancer cells‐recipient cells; *in vitro* and *in vivo*	Acquisition of carcinoma‐associated mesenchymal stromal cell‐derived mitochondria preferentially rescued mitochondrial‐poor ovarian cancer cells, increased oxidative phosphorylation and proliferation, reduced chemotherapy sensitivity, increased tumour cell heterogeneity, and promoted metastasis. Mitochondrial transfer was required for these effects and was reduced by MIRO1 knockdown in carcinoma‐associated mesenchymal stromal cells
**Nonmalignant**							
(Koyanagi *et al*., 2005) [33]	2005	Nanotubes	Nanotube‐like connection‐mediated mitochondrial transfer	Cardiomyocytes‐to‐endothelial progenitor cells	Cardiovascular/noncancer	Neonatal rat cardiomyocytes‐donor cells; human endothelial progenitor cells‐recipient cells	Adult circulating blood‐derived endothelial progenitor cells were shown to transiently establish ultrafine intercellular connections with neonatal rat cardiomyocytes, enabling intercellular transfer of mitochondrial material
(Li *et al*., 2014) [121]	2014	TNTs	TNT‐mediated mitochondrial transfer	iPSC‐derived mesenchymal stem cells‐to‐airway epithelial cells	Lung / noncancer	Human induced pluripotent stem cell‐derived mesenchymal stem cells – donor cells; bone marrow‐derived mesenchymal stem cells‐donor cells; cigarette smoke‐exposed airway epithelial cells‐ recipient cells	Acquisition of iPSC‐ mesenchymal stem cell‐derived mitochondria preserved ATP content, rescued cigarette smoke‐induced mitochondrial dysfunction in airway epithelial cells, and attenuated lung damage and fibrosis. Mitochondrial transfer occurred through TNTs and was more efficient than transfer from bone marrow‐derived MSCs
(Jackson *et al*., 2016) [122]	2016	TNTs	TNT‐mediated mitochondrial transfer	Mesenchymal stem cells‐to‐macrophages	Lung / Acute Respiratory Distress Syndrome / noncancer	Mesenchymal stem cells – donor cels; macrophages/innate immune cells‐recipient cells; *in vitro* and *in vivo* models of ARDS	Acquisition of mesenchymal stem cell‐derived mitochondria by macrophages enhanced bioenergetics and phagocytosis *in vitro* and *in vivo*, revealing mitochondrial transfer via TNTs as an important mechanism underlying the antimicrobial effects of mesenchymal stem cells in ARDS
(Hayakawa *et al*., 2016) [110]	2016	EVs	Extracellular mitochondrial particle‐mediated mitochondrial transfer	Astrocytes‐to‐neurons	Nervous system/Strokes/noncancer	Primary astrocytes‐donor cells; primary neurons from cerebral cortices‐recipient cells	Acquisition of astrocyte‐derived mitochondria increased neuronal ATP levels, enhanced cell‐survival signalling, improved neuronal viability after ischaemic stress, and contributed to neuroprotection after stroke. CD38 signalling may facilitate mitochondrial transfer from astrocytes to neurons, thereby supporting neuronal survival and plasticity after injury
(Morrison *et al*., 2017) [124]	2017	EVs	EV‐mediated mitochondrial transfer	Mesenchymal stromal cells‐to‐macrophages	Lung/Acute Respiratory Distress Syndrome/noncancer	Human mesenchymal stromal cells – donor cells; human monocyte‐derived macrophages and murine alveolar macrophages ‐recipient cells; clinically relevant models of acute lung injury/ARDS, including *in vitro*, *ex vivo* and *in vivo* systems	Mesenchymal stromal cell‐derived extracellular vesicles promoted an anti‐inflammatory, highly phagocytic macrophage phenotype through mitochondrial transfer, enhanced macrophage oxidative phosphorylation, and contributed to the amelioration of lung injury
(Silva *et al*., 2021) [125]	2021	EVs	EV‐mediated mitochondrial transfer	Mesenchymal stromal cells‐to‐airway epithelial and endothelial cells	Lung/Acute Respiratory Distress Syndrome/noncancer	Human mesenchymal stromal cells‐donor cells, primary human small airway epithelial cells and pulmonary microvascular endothelial cells‐recipient cell; injured lung cells / lung tissue; *in vitro* and *in vivo*	Mesenchymal stromal cell‐derived extracellular vesicles restored normal levels of mitophagy, mitochondrial biogenesis and barrier integrity in both cells types – injured alveolar epithelial and endothelial cells. EV‐associated mitochondria were required for protection against lung injury and restoration of lung tissue mitochondrial respiration *in vivo*
(Thomas *et al*., 2022) [123]	2022	EVs	EV‐mediated mitochondrial transfer	Mesenchymal stromal cells‐to‐chondrocytes	Musculoskeletal/noncancer	Human mesenchymal stromal cells‐donor cells; chondrocytes with mitochondrial dysfunction‐recipient cells; EV isolation, conditioned transfer experiments; *in vitro*	Mesenchymal stromal cell‐derived EVs contained intact, functional mitochondria that were taken up by chondrocytes in the absence of direct cell–cell contact, thereby increasing mitochondrial content and supporting recovery from mitochondrial dysfunction
(Dave *et al*., 2023) [111]	2023	EVs	EV‐mediated mitochondrial transfer	Brain endothelial cells‐to‐brain endothelial cells	Nervous system/Strokes/noncancer	Human brain endothelial cells‐donor cells; primary human brain endothelial cells under ischaemic stress‐recipient cells; *in vitro* and *in vivo*	Medium‐to‐large EVs containing mitochondria transferred mitochondrial cargo to recipient brain endothelial cells, where it colocalised with the endogenous mitochondrial network, increased ATP levels and mitochondrial function and reduced mouse brain infarct size

The earliest TNT‐related studies established two important principles: first, mitochondria can be transported from cell to cell, and second, this transfer has functional consequences for recipient cells. Direct visual evidence came from cocultures of cardiomyocytes and endothelial progenitor cells, in which MitoTracker‐labelled mitochondria were seen moving through nanotube‐like connections, providing the first demonstration that mitochondria can traffic between mammalian cells through such structures [33]. Soon afterwards, donor mitochondria from stem or somatic cells were shown to restore aerobic respiration in mammalian recipient human A549 lung adenocarcinoma cells lacking functional mitochondria, shifting the concept of mitochondrial transfer from a descriptive observation to a biologically meaningful rescue process [103]. It has also been shown that, in neurons and along axons, mitochondria are not necessarily transported only as isolated organelles, but may also cotransport mRNA [108, 109].

A growing number of studies have demonstrated that intercellularly transferred mitochondria can functionally influence recipient cells by increasing ATP production [45, 105, 110, 111], enhancing or restoring mitochondrial respiration [103, 106, 112, 113], increasing basal respiration and spare respiratory capacity [54, 114] and promoting resistance to stress [41, 104]. In cancer, horizontal mitochondrial transfer supports tumour progression and has been associated with enhanced survival, metabolic plasticity, stress resistance, restoration of tumorigenic potential, increased migration and invasiveness, and chemoresistance [41, 45, 46, 74, 85, 104, 105, 106, 107, 113, 115, 116, 117, 118]. Intercellular mitochondrial transfer has been shown to occur between cancer cells and endothelial cells [104], mesenchymal stem cells, bone marrow stromal cells [45, 47, 105, 107], mesenchymal stem cells [103, 118], astrocytes [85, 115], carcinoma‐associated mesenchymal stem cells [73], cancer‐associated neurons [119], cancer‐associated fibroblasts [116, 117, 120] and immune cells [54, 113, 114].

Studies in nonmalignant and injury‐related systems have also shown that tunnelling nanotubes can mediate the transfer of whole mitochondria. Because TNTs provide direct physical continuity between donor and recipient cells, they offer a route for targeted and potentially directional movement of large cargoes, including organelles. In healthy or injury‐related systems, this mode of transfer is most often associated with rescue of stressed cells, restoration of mitochondrially damaged cells and enhanced phagocytic activity of immune cells [33, 121, 122]. Extending this concept into a therapeutic setting, nonmalignant bone marrow stromal cells were shown to establish nanotube‐like connections with CD8+ T cells, enabling mitochondrial transfer that enhanced recipient T‐cell metabolic fitness and antitumour activity in both murine and human systems [114].

By contrast, subsequent studies in nonmalignant tissues showed that mitochondrial transfer is not confined to TNTs and can also occur through extracellular vesicle‐associated routes. In a model of nervous system injury, astrocytes were shown to release functional mitochondria that entered neurons, where their entry amplified cell‐survival signals [110]. More recently, in a nonmalignant neurovascular model combining ischemic endothelial cultures with *in vivo* stroke experiments, brain endothelial cell‐derived extracellular vesicles were shown to deliver mitochondrial cargo to recipient endothelial cells and to reduce brain infarct size [111]. In an *in vitro* cartilage model, human mesenchymal stromal cells released extracellular vesicles containing functional mitochondria that were transferred to chondrocytes without direct cell–cell contact [123]. A similar EV‐mediated mechanism was also described in lung injury models, where MSC‐derived extracellular vesicles transferred mitochondrial cargo to macrophages and ameliorated lung injury *in vivo* [124] and to airway epithelial, pulmonary endothelial cells, as well as human precision‐cut lung slices with improved alveolar–capillary barrier properties by restoring mitochondrial function [125].

In cancer, the best‐characterised and most frequently reported route of intercellular transfer of whole mitochondria is via tunnelling nanotubes, although extracellular vesicles, cell fusion and other mechanisms have also been described. Tunnelling nanotube‐mediated transfer of functional mitochondria from untreated pheochromocytoma PC12 cells rescued UV‐stressed PC12 cells in the early stages of apoptosis [41]. In cancer‐related settings, this concept was subsequently extended to interactions between stromal and malignant cells. Endothelial cells were shown to transfer mitochondria and other cytoplasmic material to breast cancer cells, thereby altering recipient cell phenotype and stress resistance [104], while cancer‐associated fibroblasts donated mitochondria that increased mitochondrial ATP production and promoted cancer cell migration in 3D spheroid models [116]. Similar observations have been made in hematological malignancies, where bone marrow stromal cells donated functional mitochondria to acute myeloid leukaemia cells and to primary multiple myeloma cells, promoting bioenergetic plasticity and metabolic adaptation [47, 107]. In line with this, enhanced acquisition of mitochondria by myeloma cells from bone marrow stromal cells after chemotherapeutic stress was associated with increased ATP levels, reduced mitochondrial superoxide and increased drug resistance [45].

Beyond stromal support, mitochondrial transfer has also emerged as a mechanism through which tumour cells exploit other components of the microenvironment. Recipient cancer cells acquiring mitochondria from immune cells via TNTs displayed increased basal respiration and spare respiratory capacity, whereas both parameters were reduced in donor immune cells [54]. *In vivo* experiments confirmed horizontal transfer of mitochondria from host immune cells to melanoma cells, which enhanced oxidative phosphorylation in melanoma cells and promoted lymph node metastasis, while the loss of mitochondria in immune cells caused a widespread impairment of immune surveillance [113]. Notably, mitochondrial exchange is not unidirectional. Cangkrama and co‐authors described the reverse transfer route, showing that vulvar carcinoma cells donate mitochondria to skin fibroblasts in cocultures and xenograft tumours, thereby inducing cancer‐associated fibroblast differentiation. In recipient fibroblasts, this was accompanied by metabolic reprogramming, including increased basal respiration, proton leak, oxidative phosphorylation and ATP production, and was dependent on the mitochondrial trafficking protein Miro2/RHOT2 [74]. Together, these studies indicate that mitochondrial transfer can reshape both tumour cells and stromal cells in ways that favour tumour progression. This functional relevance is further supported by studies linking mitochondrial transfer to invasive behaviour, tumour‐promoting signalling and adaptation to hostile microenvironments. In bladder cancer, mitochondrial transfer activated AKT/mTOR signalling and increased invasiveness both *in vitro* and *in vivo* [46]. In glioblastoma, TNT‐mediated mitochondrial transfer enables astrocytes to adapt to hypoxic conditions, modifying the microenvironment to support tumour invasion [85]. Likewise, horizontal mitochondrial transfer from astrocytes was shown to promote cell‐cycle progression to the proliferative G2/M phase and to enhance self‐renewal and tumorigenicity in glioblastoma cells [115]. Patient‐derived glioblastoma models also form TNTs and TM in organoids, facilitating mitochondrial transfer, which may contribute to progression and treatment resistance [7].

Although TNTs represent the most extensively characterised route for transfer of whole mitochondria, extracellular vesicles constitute a second important pathway of mitochondrial exchange in cancer. In hormonal therapy‐resistant breast cancer, CAF‐derived extracellular vesicles carrying the full mitochondrial genome were shown to transfer mtDNA to hormone therapy‐naive and metabolically dormant breast cancer cells, thereby restoring oxidative phosphorylation, promoting escape from metabolic quiescence and contributing to endocrine therapy resistance, particularly in cancer stem‐like populations [117]. In lung cancer, extracellular vesicles released by tumour cells and carrying miR‐1290 activated normal fibroblasts into CAFs via the MT1G/AKT pathway, thereby promoting metastasis; these activated CAFs subsequently transferred mtDNA to tumour cells with damaged mitochondria, restoring mitochondrial function and enhancing proliferation, migration and epithelial–mesenchymal transition [120].

A third mechanism of intercellular mitochondrial transport is direct cell–cell contact, as demonstrated in a coculture system in which mitochondria from carcinoma‐associated mesenchymal stromal stem cells stably expressing COX8–GFP were transferred to ovarian cancer cells, thereby restoring metabolic fitness and promoting the proliferation and chemoresistance of mitochondria‐poor tumour cells [73]. In parallel, several earlier studies demonstrated the acquisition of mitochondrial genomes or whole mitochondria by cancer cells even when the exact transport route remained unresolved. Metastatic murine B16 melanoma and 4T1 breast carcinoma cell models lacking mitochondrial DNA were shown to exhibit slower tumour growth *in vivo*; however, after acquiring mtDNA and whole mitochondria from host cells within the TME, mitochondrial respiration was restored, providing evidence for horizontal mitochondrial genome transfer [106, 112]. Similarly, bone marrow stromal cells were shown to donate functional mitochondria to acute myeloid leukaemia cells during chemotherapy, with evidence supporting involvement of an endocytic uptake pathway; this transfer increased mitochondrial mass and ATP production and contributed to drug resistance in leukemic progenitors [105].

Taken together, these studies identify intercellular mitochondrial transfer as a functionally important mode of communication in cancer. Across solid tumours and hematological malignancies, it supports metabolic plasticity, stress adaptation, invasion and treatment resistance. Although TNT‐mediated transfer of whole mitochondria remains the best‐characterised route, vesicle‐mediated and other mechanisms further broaden the spectrum of mitochondrial exchange within the tumour microenvironment, highlighting this process as a potentially targetable feature of cancer biology.

## Horizontal transfer of RNA and ribosomes in cancer

Beyond mitochondria, TNTs have also been shown to mediate the intercellular transfer of nucleic acids and components of the protein translation machinery. Recent studies indicate that TNT‐mediated mRNA transfer can alter the state of recipient cells, with one report demonstrating transcriptomic reprogramming in human pluripotent stem cells [25] and another showing that transferred mRNAs can be translated in recipient cells and functionally rescue specific genetic defects *in vitro* [96].

Evidence for the transfer of ribosome‐related components has been reported in several systems. Early observations showed that Schwann cells can deliver ribosomes to desomatised axons, where Schwann cell protrusions invaginate into the axon and release ribosomes into the axoplasm [126]. In line with this concept, ribosomal proteins were subsequently detected in protrusions from hydrogen peroxide‐treated CAD cells [93], while proteomic analysis of TNTs in U2OS osteosarcoma cells identified numerous ribosomal proteins in addition to tetraspanins CD9 and CD81 [52]. The functional significance of TNT‐mediated mRNA transfer was demonstrated by evidence that PEX6 mRNA transferred via direct cell–cell contact was translated in recipient cells, resulting in *de novo* peroxisome biogenesis in PEX6‐mutant fibroblasts cocultured with wild‐type cells, and thereby complementing the mutant phenotype through mRNA transfer [96]. A broader biological relevance of this phenomenon is supported by the finding that direct cell contact‐mediated coculture with mouse embryonic stem cells promotes conversion of human primed pluripotent stem cells to a naïve‐like state through transfer of mouse‐derived mRNAs, with *Tfcp2l1*, *Tfap2c*, and *Klf4* identified as indispensable for this process [25]. The concept is further supported by work showing that intercellular mRNA transfer may also occur *in vivo* in human‐to‐mouse xenograft models [127].

Tunnelling nanotubes between pancreatic cancer cells were shown to mediate the transfer of polyadenylated mRNAs, 5.8S rRNA, ribosomal proteins, and fully assembled ribosomes. Coculture of translation‐impaired recipient cells with translationally competent donor cells partially restored protein synthesis in the affected cells through ribosome transfer, whereas donor cells displayed a concomitant reduction in their own protein synthesis [28]. This reciprocal gain–loss relationship strongly mirrors the transport asymmetry described by Saha and co‐authors, where the mitochondrial transfer from immune cells increased the basal respiration and spare respiratory capacity in cancer cells but diminished these parameters in the donor immune cells [54]. It remains unclear whether ribosomes are transferred as monosomes, polysomes, in association with other organelles, or in some cases simply accompany transported mRNAs. The concept of possible cotransport is supported by studies showing that Pink1 mRNA is cotransported with mitochondria in neurons [108] and that nuclear‐encoded Cox7c mRNA is likewise associated with mitochondria in a mouse neuronal cell line and within axons of primary mouse motor neurons [109].

These findings add support to the hypothesis that the TNT‐mediated transfer of mRNAs and ribosomes represents a novel form of intercellular cooperation/parasitism by dynamically redistributing metabolic and proteosynthetic capacity. Since ribosomal biogenesis and proteosynthetic capacity have been suggested as key parameters of cancer spread and resilience [128, 129], these recent discoveries open new avenues for exploring how cancer cells adapt to stress, nutrient deprivation and therapeutic pressure, linking a translational reprogramming via mRNA and ribosome exchange to the well‐established paradigm of mitochondrial transfer and metabolic cooperation within the tumour microenvironment.

## Future research and its challenges

In this review, we summarised the current knowledge of TNTs, including their significance, structure, induction and the molecular mechanisms underlying intercellular transfer, with particular emphasis on mitochondria, mRNAs and ribosomes. Available studies indicate that TNTs are dynamic intercellular conduits capable, through transferred material, of altering the stress adaptation, treatment responsiveness, metabolic state of connected cells, as well as rescuing specific genetic defects and reprogramming cells. In cancer, these processes may promote tumour progression by supporting bioenergetic and translational rescue and adaptation to microenvironmental and therapeutic stress.

Among cargoes transferred via TNTs, mitochondrial transfer is currently the best‐established example of functionally significant organelle exchange. Work in both nonmalignant and malignant systems shows that transferred mitochondria can restore oxidative phosphorylation, increase ATP production, alter migration and invasion, and influence sensitivity to treatment. However, there remains considerable scope for further research better to understand the signalling pathways and the mechanistic basis of TNT‐mediated transport. The specific signals and mechanisms that induce TNT formation under stress or nutrient deprivation are largely unknown, as are potential strategies for the inhibition of such putative signals. Proteins required for the attachment of a growing TNT to the target cell and those responsible for the fusion of the two membranes are yet to be sufficiently characterised. The ability to manipulate the stimuli for TNT formation or the specific connections they create would be of great interest not only in research but, potentially, also in cancer treatment.

Recent studies further suggest that TNT‐mediated transfer is not limited to organelles such as mitochondria, but can also involve mRNAs, rRNA, ribosomal proteins and fully assembled ribosomes, raising the possibility that TNTs redistribute proteosynthetic capacity between cells. This has important conceptual implications, because it suggests that stressed or translation‐impaired cells may be rescued not only metabolically, but also at the level of protein synthesis, through functional complementation of specific defects and even cell‐state reprogramming. These findings open an important new area of research, but many fundamental questions remain unresolved, including whether ribosomes are transferred as monosomes, polysomes, in association with other organelles, or just simply accompany transported mRNA.

A major challenge for future work is the limited availability of cargo‐specific experimental tools. It is important to note that MitoTracker dyes may exhibit nonspecific partial enrichment in structures other than mitochondria, thereby complicating the interpretation of horizontal mitochondrial transfer. Consistent with this, red blood cells, which naturally lack mitochondria, were nevertheless shown to transfer the MitoTracker signal to recipient cells, indicating that dye transfer does not necessarily correspond to the transfer of intact mitochondria [130]. Similarly, another group of authors showed that MitoTracker is rapidly transferred from both astrocytes and astrocyte‐conditioned media to neurons independently of mitochondrial transfer [131]. We observed a similar phenomenon in our own experiments, in which carboxyfluorescein succinimidyl ester (CFSE), a dye that covalently labels intracellular proteins by reacting with amine groups and is often used in coculture to distinguish donor and recipient cell populations, was partially transferred via TNTs to previously unstained recipient cells [28]. These observations suggest that caution is warranted when interpreting experiments on horizontal intercellular transfer involving dyes such as MitoTracker, and possibly also CFSE, as their signal may not always exclusively reflect the transfer of the intended cargo or reliably distinguish the cell population of interest. In the mRNA field, for example, widely used MS2‐based approaches can themselves interfere with TNT‐mediated transfer, complicating interpretation of trafficking experiments [96]. To investigate ribosomes, a newly developed selective fluorescent probe RiboBright for eukaryotic ribosomes shows promise for the monitoring of cellular organisation and ribosome dynamics [132].

From a therapeutic perspective, TNT‐mediated cargo exchange presents both an opportunity and a challenge. In regenerative or genetic disease contexts, intercellular transfer of mitochondria or mRNAs may offer new strategies for functional rescue. In cancer, however, the same processes are more likely to support tumour cell survival, metabolic flexibility and resistance to therapy. While a complete inhibition of these processes—whether through the disruption of actin or microtubule dynamics, inhibition of motor proteins or modulation of membrane attachment and fusion—is unlikely to provide a sufficient therapeutic window because of the known general toxicity of such inhibitors, targeting specific proteins involved only or predominantly in TNT‐related processes may offer novel therapeutic avenues for modulating intercellular communication, cooperation or parasitism within the tumour microenvironment. Just a little over 20 years after the discovery of TNTs, the field is still in its infancy and we strongly believe it promises many rewarding discoveries.

## Conflict of interest

The authors declare no conflict of interest.
